# P-2195. Hepatitis C Virus Screening Guidelines Rates Among a National Sample

**DOI:** 10.1093/ofid/ofae631.2349

**Published:** 2025-01-29

**Authors:** Kenta Ferval-Shioya, David C Kaelber, Allan M Kerandi

**Affiliations:** Mamaroneck High School, Mamaroneck, New York; MetroHealth Medical Center/ Case Western Reserve University, Cleveland, Ohio; MetroHealth/University Hospitals/CWRU, Cleveland, Ohio

## Abstract

**Background:**

Objective:

Evaluate US hepatitis C virus (HCV) screening rates after the 2013 and 2020 US Preventive Services Task Force HCV screening recommendations.

Design/Participants:

A retrospective observational study utilizing TriNetX, an electronic health record (EHR) aggregation platform among 100 million patients.

**Table 1.**
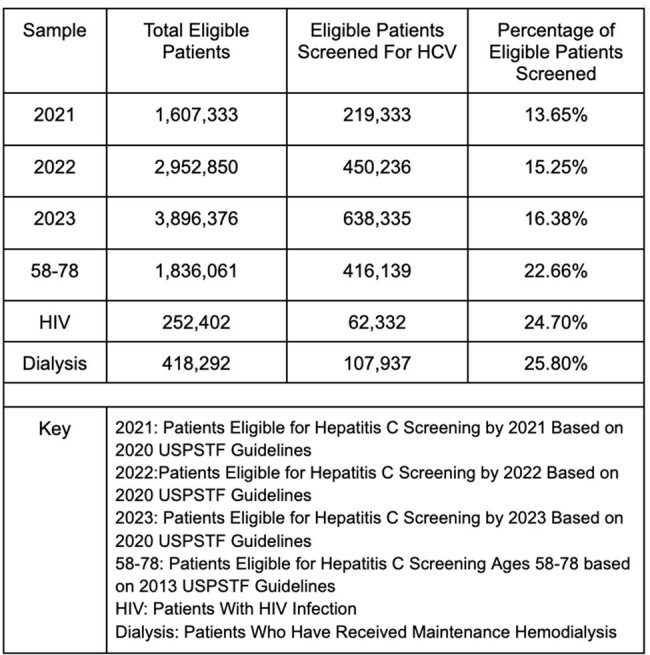
Percentages of Patients Screened for Hepatitis C Based on Sample

**Methods:**

We identified patients eligible for HCV screening based on their age (58-78 for 2013 guidelines and 18-79 for 2020 guidelines) and having a preventative care visit, based on common procedure terminology (CPT) codes. Successful screening encompassed a HCV antibody test result. High risk patients: encounter diagnosis ICD codes for HIV or renal dialysis or a CPT code for hemodialysis. We examined age distribution, gender, race, and ethnicity for possible screening disparities.

**Figure 1.**
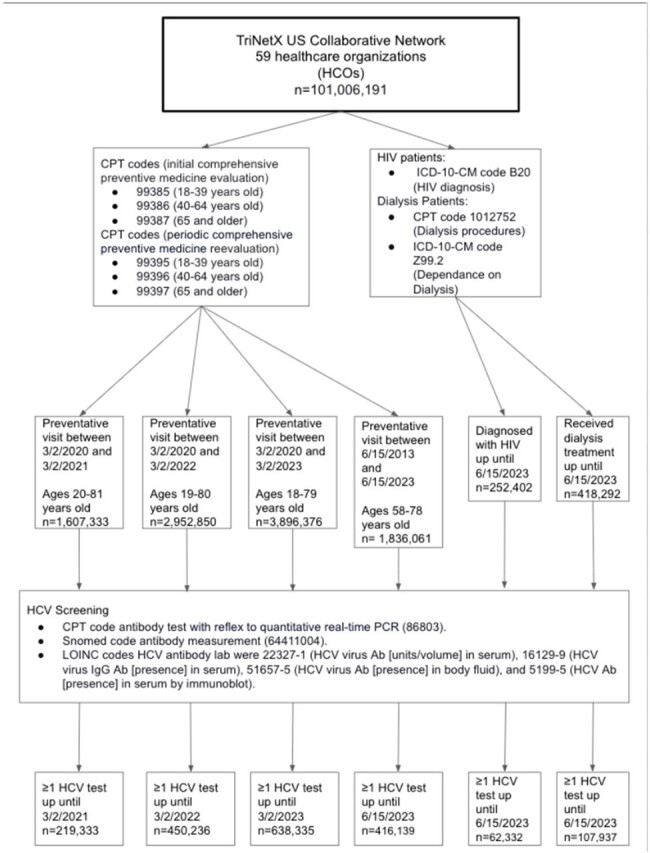
Overview of Cohorts.

**Results:**

In 2021, the first year after 2020 guidelines, 13.65% (219,333/1,607,333) of patients were screened. In 2022 and 2023, 15.25% (450,236/2,952,850) and 16.38% (638,335/3,896,376) were screened. In 2023, ten years after the 2013 guidelines, 22.66% (416,139/1,836,061) of eligible patients were screened. Among high-risk patients 10-year after the 2013 guidelines, 24.70% (62,332/252,402) with HIV and 25.80% (107,937/418,292) on dialysis were screened.

In our disparities analysis, we found that the percentage of unknown ethnicity or race, Asian, or other races was significantly less (p-value < .001) among screened eligible patients compared to eligible patients based on the 2020 and 2013 guidelines.

Over time screening disparities generally decreased so that people eligible based on the 2013 guidelines had fewer disparities in 2023 than those eligible based on the 2020 guidelines, except for gender where disparities increased between the two sets of guidelines with men being screened ∼3% more than women based on the 2013 guidelines.

**Figure 2.**
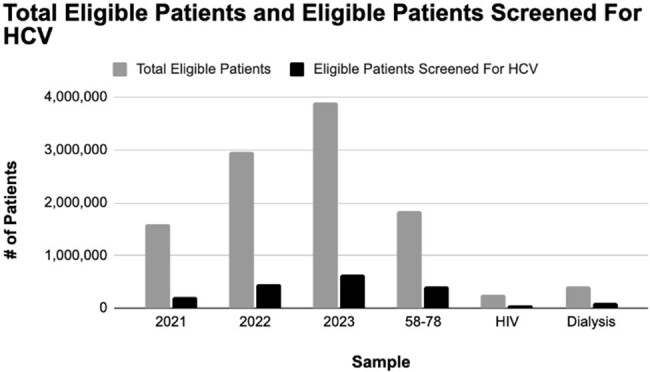
Total Eligible Patients and Eligible Patients Screened for HCV.

**Conclusion:**

The current percentage of eligible patients screened for hepatitis C (< 20%) based on current guidelines is low and is only increasing slowly (1-2% per year). Even high-risk patients were only screened ∼25% of the time.

**Figure 3.**
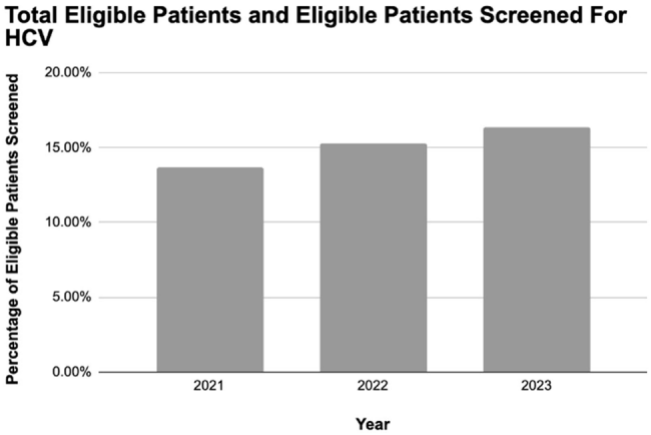
Percentage of Eligible Patients Screened for HCV.

**Disclosures:**

David C. Kaelber, MD, PhD, MPH, FAAP, FACP, FACMI, FAMIA, Dynavax Technologies Corporation: Advisor/Consultant

